# Ligand Binding and Activation of PPAR**γ** by Firemaster^®^ 550: Effects on Adipogenesis and Osteogenesis *in Vitro*

**DOI:** 10.1289/ehp.1408111

**Published:** 2014-07-25

**Authors:** Hari K. Pillai, Mingliang Fang, Dmitri Beglov, Dima Kozakov, Sandor Vajda, Heather M. Stapleton, Thomas F. Webster, Jennifer J. Schlezinger

**Affiliations:** 1 Department of Environmental Health, Boston University, Boston, Massachusetts, USA; 2 Nicholas School of the Environment, Duke University, Durham, North Carolina, USA; 3 Department of Biomedical Engineering, Boston University, Boston, Massachusetts, USA

## Abstract

Background: The use of alternative flame retardants has increased since the phase out of pentabromodiphenyl ethers (pentaBDEs). One alternative, Firemaster® 550 (FM550), induces obesity in rats. Triphenyl phosphate (TPP), a component of FM550, has a structure similar to that of organotins, which are obesogenic in rodents.

Objectives: We tested the hypothesis that components of FM550 are biologically active peroxisome proliferator-activated receptor γ (PPARγ) ligands and estimated indoor exposure to TPP.

Methods: FM550 and its components were assessed for ligand binding to and activation of human PPARγ. Solvent mapping was used to model TPP in the PPARγ binding site. Adipocyte and osteoblast differentiation were assessed in bone marrow multipotent mesenchymal stromal cell models. We estimated exposure of children to TPP using a screening-level indoor exposure model and house dust concentrations determined previously.

Results: FM550 bound human PPARγ, and binding appeared to be driven primarily by TPP. Solvent mapping revealed that TPP interacted with binding hot spots within the PPARγ ligand binding domain. FM550 and its organophosphate components increased human PPARγ1 transcriptional activity in a Cos7 reporter assay and induced lipid accumulation and perilipin protein expression in BMS2 cells. FM550 and TPP diverted osteogenic differentiation toward adipogenesis in primary mouse bone marrow cultures. Our estimates suggest that dust ingestion is the major route of exposure of children to TPP.

Conclusions: Our findings suggest that FM550 components bind and activate PPARγ. In addition, *in vitro* exposure initiated adipocyte differentiation and antagonized osteogenesis. TPP likely is a major contributor to these biological actions. Given that TPP is ubiquitous in house dust, further studies are warranted to investigate the health effects of FM550.

Citation: Pillai HK, Fang M, Beglov D, Kozakov D, Vajda S, Stapleton HM, Webster TF, Schlezinger JJ. 2014. Ligand binding and activation of PPARγ by Firemaster® 550: effects on adipogenesis and osteogenesis *in vitro*. Environ Health Perspect 122:1225–1232; http://dx.doi.org/10.1289/ehp.1408111

## Introduction

Flammability standards, such as California’s Technical Bulletin 117 (Bureau of Home Furnishings and Thermal Insulation 2000), led to the addition of flame retardants at 2–5% levels in residential furniture, making these chemicals a ubiquitous component of the human indoor environment. The U.S. production phase-out of commercial pentabromodiphenyl ethers (pentaBDEs) in 2004 led to an increased demand for alternatives such as organophosphate flame retardants (OPFRs) and a commercial mixture known as Firemaster® 550 (FM550). FM550 is composed of bis-(2-ethylhexyl) tetrabromophthalate (TBPH), a brominated analogue of bis-(2-ethylhexyl) phthalate (a known obesogen) (Feige et al. 2010); tetrabromobenzoate (TBB); and a mixture of triaryl phosphates including triphenyl phosphate (TPP) and several isomers of mono-, di-, and triisopropylated triaryl phosphates (ITPs). FM550 is composed of approximately 40% of the brominated components and 60% of the organophosphate components (Stapleton et al. 2008). High concentrations of OPFRs have been found in dust collected from homes, offices, and cars, with concentrations of TPP alone as high as 1.8 mg/g house dust (Carignan et al. 2013; Meeker et al. 2013; Stapleton et al. 2009). OPFR metabolites are ubiquitous in human urine (Carignan et al. 2013; Cooper et al. 2011; Meeker et al. 2013).

Patisaul et al. (2013) reported that prenatal and postnatal exposure to FM550 resulted in increased anxiety, obesity, and early puberty in rats, with the nonbehavioral effects occurring at a dose of 1 mg per pregnant rat per day or approximately 2.5 mg FM550/kg body weight (BW)/day. Because TPP represents 10–20% of the FM550 mixture, this equates to approximately 250 μg TPP/kg BW/day. These data suggest that a component, or components, of FM550 is acting as an environmental obesogen, a chemical that disrupts the homeostatic controls of adipogenesis and energy balance (Grun et al. 2006). Similarly, increased adiposity has been observed in rodents exposed to tributyltin and phthalates (Feige et al. 2010; Grun et al. 2006). These obesogens are agonists of peroxisome proliferator-activated receptor γ (PPARγ) (Feige et al. 2007; Grun et al. 2006), a nuclear receptor and master regulator of adipogenesis (Tontonoz and Spiegelman 2008). Interestingly, TPP has been shown to interact with a variety of nuclear receptors, activating constitutive androstane receptor, pregnane X receptor, and estrogen receptor–mediated reporter activity and antagonizing androgen, progesterone, and glucocorticoid receptor–mediated reporter activity (Honkakoski et al. 2004; Kojima et al. 2013; Suzuki et al. 2013). The ability of TPP to interact with PPARγ remains to be elucidated.

On the basis of the profound adiposity induced by FM550 *in vivo* and evidence that OPFRs can act as nuclear receptor ligands, we designed experiments to test the hypothesis that components of FM550 are biologically active PPARγ ligands. Accordingly, we examined FM550, as well as the components TPP and ITP, for the ability to bind PPARγ, to initiate PPARγ-dependent transcription, to induce mature adipocyte differentiation, and to divert bone marrow multipotent mesenchymal stromal cell (MSC) differentiation away from osteogenesis. We also estimated the potential contribution of dust ingestion to TPP exposure based on previous measurements in dust and a screening-level exposure model for semivolatile organic compounds (SVOCs). Overall, the data presented here are consistent with FM550 containing a PPARγ ligand and with TPP being a major contributor to PPARγ activation. TPP not only induced adipocyte differentiation but also antagonized osteogenesis in primary mouse bone marrow cultures.

## Materials and Methods

*Materials*. Human insulin, Nile red, tributyltin chloride (96%), and TPP (≥ 99%) were from Sigma-Aldrich (St. Louis, MO). We purchased a mixture of ITP (commercial grade) from the Chinese manufacturer Jinan Great Chemical Industry Co. Ltd (Jinan, People’s Republic of China). FM550 was a gift from Chemtura Inc. (Gastonia, NC) to S. Klosterhaus (Stapleton et al. 2008). TBPH [99.5% by gas chromatography/mass spectrometry (GC/MS)] and TBB (97.3% by GC/MS) were from AccuStandard (New Haven, CT). All other reagents were from Thermo Fisher Scientific (Suwanee, GA), unless noted.

*Preparation of dose solutions and estimation of mixture molar concentrations*. Dose solutions were prepared in DMSO based on weight per volume for FM550 and ITP, and on a molar basis for TPP, TBB, TBPH, and rosiglitazone. Molecular weights for the FM550 and ITP mixtures were estimated based on the molecular weights of the individual components and their typical percentages in these mixtures (see Supplemental Material, Table S1), and molar concentrations for the experiments were estimated using the calculated molecular weights (see Supplemental Material, Table S2). For FM550 and ITP, the concentrations are reported as weight per volume, followed by the estimate of the molar concentration.

*PPAR*γ *ligand binding assay*. Human PPARγ binding was quantified using the PolarScreen^TM^ PPARγ-Competitor Assay Kit (Invitrogen, Carlsbad, CA), according to the manufacturer’s instructions. In brief, human recombinant PPARγ ligand binding domain (LBD)–glutathione *S*-transferase and Fluormone™ PPARγ green, a tight-binding, selective, fluorescent PPARγ ligand, were mixed with test compounds. FM550 (0.01–70 μg/mL; 0.02–160 μM), TPP (0.01–1,400 μM), TBB (0.009–90 μM), TBPH (0.12–1200 μM), ITP (0.01–28 μg/mL; 0.02–80 μM), and the positive control rosiglitazone (0.00012–12 μM) were prepared in DMSO. Displacement of the fluorescent ligand, which has high polarization when bound to the PPARγ-LBD and low polarization when not bound, was assessed by measuring loss of fluorescence polarization using a SpectraMax M5 plate reader (Molecular Devices, Sunnyvale, CA). We calculated the IC_50_ (concentration required to reduce effect by 50%) and dissociation constants to compare the potency of the binding. Dissociation constants were calculated according to the following equation:

IC_50_/[PPARγ green] = *K*_d,ligand_/*K*_d,probe_ [1],

where *K*_d,probe_ is the dissociation constant calculated from titration of 1.25 nM Fluormone™ PPARγ green with the PPARγ LBD.

*Computational analysis of ligand binding to PPAR*γ. We determined binding hot spots in the PPARγ LBD using the computational solvent mapping algorithm FTMap (Brenke et al. 2009). The docking of ligands was carried out using the docking program AutoDock Vina 1.1.0 (Trott and Olson 2010). The 10 lowest energy binding poses were retained for each ligand. The selection of the most likely pose was based on the atom densities calculated from the mapping results. We considered each retained ligand pose separately and summed the atomic densities for all heavy atoms, resulting in a measure of overlap between the pose and the probe density. The poses were ranked on the basis of this overlap measure, and the pose with the best overlap was selected as the most likely binding mode (Kozakov et al. 2011) (for a detailed description of this analysis, see Supplemental Material, “Computational analysis of ligand binding to PPARγ”).

*Reporter assays*. Cos-7 cells were transiently transfected with vectors containing human *PPARG1* (provided by V.K. Chatterjee, University of Cambridge, Cambridge, UK) (Gurnell et al. 2000) and human *RXRA* (plasmid 8882; Addgene, Cambridge, MA) (Tontonoz et al. 1994), with PPRE x3-TK-luc (plasmid 1015; Addgene) (Kim et al. 1998) and CMV-eGFP reporter constructs using Lipofectamine2000 (Invitrogen). Cultures were cotransfected with either pcDNA3 (Invitrogen) or dominant negative human *PPARG* (PPARγ-DN; provided by V.K. Chatterjee). Following an overnight incubation, the medium was replaced, and cultures received no treatment (naive) or were treated with vehicle (DMSO, 0.1%), FM550 (0.1–20 μg/mL; 0.2–50 μM), TPP (0.1–40 μM), ITP (0.1–10 μg/mL; 0.3–60 μM), or rosiglitazone (0.0001–1 μM). After 24 hr incubation, cells were lysed in Glo Lysis Buffer and mixed with Bright Glo reagent (both from Promega, Madison, WI). Luminescence and fluorescence were determined using a Synergy2 plate reader (Biotek, Inc., Winooski, VT). Luminescence was normalized by GFP (green fluorescent protein) fluorescence in the same well. The normalized luminescence for each well was then divided by the normalized luminescence measured in control DN-PPARγ–transfected wells to determine the fold-change from DN-control.

*Cell culture*. BMS2 cells are C57BL/6 mouse–derived bone marrow stromal cells (provided by P. Kincade, Oklahoma Medical Research Foundation, Oklahoma City, OK) (Pietrangeli et al. 1988). BMS2 cells were maintained in Dulbecco’s modiﬁed Eagle’s medium (DMEM) with 5% fetal bovine serum (FBS) (Sigma-Aldrich), 5 μg/mL plasmocin (Invivogen, San Diego, CA), and 20 mM l-glutamine. Cultures were maintained at 37°C in a humidified 5% CO_2_ atmosphere. BMS2 cells were plated at 40,000 cells/well (24-well plates) or 160,000 cells/well (6-well plates) in preadipocyte medium (DMEM containing 10% FBS, 1 mM sodium pyruvate, 100 U/mL penicillin, and 100 μg/mL streptomycin) and allowed to become confluent (3–4 days). Prior to dosing, the medium was replaced with preadipocyte medium supplemented with insulin (0.5 μg/mL). Cultures received no treatment (naive) or were treated with vehicle (DMSO, 0.1%), FM550 (0.1–10 μg/mL; 0.2–20 μM), ITP (0.1–10 μg/mL; 0.3-30 μM), TPP (0.1–20 μM), or rosiglitazone (0.001–1 μM). Medium was changed, and the cultures were redosed once. The total exposure period was 7 days.

Primary bone marrow cultures were prepared from C57BL/6J mice (female, 12 weeks of age; Jackson Laboratories, Bar Harbor, ME). Experimental protocols were reviewed and approved by the Institutional Animal Care and Use Committee at Boston University. All animals were treated humanely and with regard for alleviation of suffering. Mice were housed four per cage, with a 12-hr light cycle. Water and food (2018 Teklad Global 18% Protein Rodent Diet, Irradiated; Harlan Laboratories, Indianapolis, IN) were provided *ad libitum*. Two days after arrival, animals were euthanized for collection of bone marrow. Limbs were aseptically dissected, and soft tissue was removed from the bone. Bone marrow was flushed from the femur, tibia, and humerus using a 25-gauge needle and RPMI media containing 10% FBS, 100 U/mL penicillin, 100 μg/mL streptomycin, and 0.25 μg/mL amphotericin B; strained through a 70-μm cell strainer; diluted in MSC media [α-MEM (α-minimal essential medium) containing 10% FBS, 100 U/mL penicillin, 100 μg/mL streptomycin, 0.25 μg/mL amphotericin B]; and seeded at 6 × 10^6^/mL in 1 mL/well in a 12-well plate or 2 mL/well in a 6-well plate. Half of the medium was replaced 4 days after plating, and the cultures continued for 3 more days. To induce osteogenesis, the medium was replaced with MSC media supplemented with ascorbate (12.5 μg/mL), β-glycerol phosphate (8 μM), dexamethasone (10 nM), and insulin (500 ng/mL). Cultures received no treatment (naive) or were treated with vehicle (DMSO, 0.1%), FM550 (0.1–10 μg/mL; 2–20 μM), TPP (0.1–10 μM), or rosiglitazone (0.1 μM). Medium was changed, and the cultures were redosed three times for a total exposure period of 7 days (gene expression) or four times for a total exposure period of 12–13 days (phenotype).

*Cell viability assays*. Confluent BMS2 cultures received no treatment (naive) or were treated with vehicle (DMSO, 0.1%), FM550 (0.1–40 μg/mL; 0.2–90 μM), TPP (0.1–40 μM), ITP (0.1–40 μg/mL; 0.3–100 μM), or rosiglitazone (0.001–1 μM) for 24 hr, 7 days, or 12 days. Treatment with high-dose tributyltin (1–4 μM) for 2–3 hr was used as a positive control. Medium was changed, and the cultures were redosed as described under “Cell Culture.” Cellularity was assessed by 3-[4,5-dimethylthiazol-2-yl]-2,5-diphenyltetrazolium bromide (MTT) labeling for 3 hr by standard methods. Apoptosis and necrosis were assessed by caspase-3 activity (Caspase-Glo® 3/7 Assay; Promega) and dead cell protease release (CytoTox-Glo™ Cytotoxicity Assay; Promega), respectively, according to the manufacturer’s instructions. Absorbance and luminescence measurements were determined using a Synergy2 plate reader. Absorbance or luminescence in experimental wells was normalized by dividing by the absorbance or luminescence measured in untreated cultures, and is reported as “fold change from medium.”

*Adipogenesis and osteogenesis assays*. Lipid accumulation was quantified in bone marrow cultures and BMS2 cells by Nile red staining (Yanik et al. 2011). The fluorescence in all experimental wells was normalized by subtracting the fluorescence measured in untreated cultures and reported as naive-corrected relative fluorescence units (RFUs). After Nile Red staining in bone marrow cultures, they were fixed in 2% paraformaldehyde. To quantify alkaline phosphatase activity, cells were incubated with *p*-nitrophenyl phosphate solution (Sigma-Aldrich). After quenching with sodium hydroxide, absorbance (405 nM) was measured. Bone marrow cultures then were stained with Alizarin red (Osteogenesis Quantitation Kit; Millipore, Billerica, MA), and staining was quantified according to the manufacturer’s instructions. For osteogenesis assays, absorbance in experimental wells was normalized by dividing by the absorbance measured in untreated osteogenic cultures and reported as fold change from medium. All absorbance and fluorescence measurements were determined using a Synergy2 plate reader.

*Immunoblotting*. Cells were lysed in Cell Lysis Buffer (Cell Signaling Technology, Beverly, MA) followed by sonication. Whole-cell lysates were used for protein expression analyses. Protein concentrations were determined by the Bradford method (Bradford 1976). Total proteins (40 μg) were resolved on 10% gels, transferred to a 0.2 μm nitrocellulose membrane, and incubated with monoclonal rabbit anti-perilipin (9349; Cell Signaling Technology). Immunoreactive bands were detected using horseradish peroxidase–conjugated secondary antibodies (Biorad, Hercules, CA) followed by enhanced chemiluminescence. To control for equal protein loading, blots were reprobed with a β-actin–specific antibody (A5441; Sigma-Aldrich) and analyzed as described above.

*mRNA expression*. Total RNA was extracted, and genomic DNA was removed using the RNeasy Plus Mini Kit (QIAGEN, Valencia, CA). cDNA was prepared from total RNA using the GoScript™ Reverse Transcription System (Promega). All quantitative reverse-transcription polymerase chain reaction (qPCR) was performed using the GoTaq® qPCR Master Mix System (Promega). The following validated primers were purchased from Qiagen: 18s ribosomal RNA (*Rn18s*; QT01036875), fatty acid binding protein 4 (*Fabp4*; QT00091532), Sp7 transcription factor 7 [Osterix (*Sp7*; QT00293181)]. qPCR was performed using a 7500 Fast Real-Time PCR System (Applied Biosystems, Carlsbad, CA). Relative gene expression was determined according to the Pfaffl method (Pfaffl 2001), with the threshold value for *Rn18s* used for normalization. No significant differences were observed in the expression of *Rn18s* across the different treatments (data not shown). The quantification cycle value from naive, undifferentiated cultures prepared from 9-week-old male mice was used as the reference point. Data are reported as fold difference from naive.

*Exposure assessment*. We used TPP dust concentrations from the largest published sample to date in which 50 homes were sampled in the Boston, Massachusetts, area between 2002 and 2007 using vacuum cleaner bags (Stapleton et al. 2009). The dust concentrations reported by Stapleton et al. (2009) are similar to those previously reported by Dodson et al. (2012) and Van den Eede et al. (2011). Because TPP is an SVOC (Weschler and Nazaroff 2008), we used the SVOC model of Little et al. (2012) to assess indoor exposure to TPP. The model estimates exposure via several routes of exposure assuming steady-state conditions: inhalation (vapor and particle bound), dermal absorption of compounds from vapor, and incidental dust ingestion. Dermal absorption from contact with dust or surface films was not included in the model of Little et al, (2012), and this is a potentially important limitation. The factor driving the model is *y*_0_, the vapor-phase concentration of the SVOC, in equilibrium with the material-phase concentration (*C*_0_) of the compound in the product. Although we previously measured *C*_0_ for TPP in furniture foam (Stapleton et al. 2012), there is no generally accepted way to estimate *y_0_* from *C_0_* for SVOCs (Little et al. 2012). However, *y_0_* can be back-calculated from the measured bulk air concentration *y* (Little et al. 2012) or, as we did, from dust concentrations (*C*_dust_) because all three are related in the steady-state model:

*y*_0_ = *y*[1 + (Q*/*hA*)] [2]

*y* = *C*_dust_/*K*_dust_, [3]

where *h* is the convective mass transfer coefficient over the emission surface, *A* is the surface area of the source (assumed to be polyurethane foam with an additive flame retardant), Q* is the equivalent ventilation rate adjusted for particulate-bound SVOCs, and *K*_dust_ is the dust/vapor partition coefficient (Little et al. 2012). These and other parameters were either taken directly from Little et al. (2012) or calculated using the physical–chemical properties of TPP. To facilitate comparisons with other compounds analyzed using this model, we estimated the physical–chemical properties of TPP using SPARC (ARChem 2013): log(*K*_OA_) of 10.3 at 25°C, log(*K*_OW_) of 7.3 at 32°C, log(*K*_WA_) of 2.8 at 32°C, where *K*_OA_ is the octanol−air partition coefficient, *K*_OW_ is the octanol−water partition coefficient, and *K*_WA_ is the water−air partition coefficient. Given the estimate of *y*_0_, the model of Little et al. (2012)—implemented in a spreadsheet provided with their publication—estimates partitioning within a room. Using standard exposure factors, the model then provides estimates of exposure via inhalation (vapor and particle bound), dermal absorption of compounds from vapor, and incidental dust ingestion.

*Statistics*. Statistical analyses and curve fitting were performed with Prism 5 (Graphpad Inc., La Jolla, CA). Data are presented as mean ± SE. The number of replicates is indicated in the figure legends. Gene expression data were log-transformed prior to analysis. Dose–response curves were fit with the sigmoid four-parameter Hill function. Data were analyzed for statistical significance using a one-factor analysis of variance (ANOVA) in conjunction with the Dunnett’s multiple comparisons test; *p* ≤ 0.05 was considered statistically significant.

## Results

*Analysis of toxicity of FM550 and its organophosphate components.* We used the BMS2 bone marrow stromal cell line to assess the toxicity of FM550, TPP, and ITP under short- and long-term dosing regimens. Confluent BMS2 cultures showed no loss of cellularity after treatment for 24 hr with concentrations as high as 40 μg/mL (90 μM) FM550, 40 μM TPP, or 40 μg/mL (100 μM) ITP or after treatment for 7 days with concentrations as high as 20 μg/mL (50 μM) FM550, 20 μM TPP, or 10 μg/mL (30 μM) ITP (see Supplemental Material, Figure S1A,B). Confluent BMS2 cultures showed no loss of cellularity, no increase in caspase-3 activity, and no increase in necrotic protein release after treatment for 12 days with concentrations as high as 10 μg/mL (20 μM) FM550 or 20 μM TPP (see Supplemental Material, Figure S1C–E). The positive control, tributyltin, showed a significant reduction in cellularity and significant increases in apoptosis and necrosis, confirming that the assays were functional (see Supplemental Material, Figure S1A–E). Rosiglitazone showed no loss of cellularity following any treatment period up to 12 days at a concentration as high as 1 μM (see Supplemental Material, Figure S1A–C).

**Figure 1:**
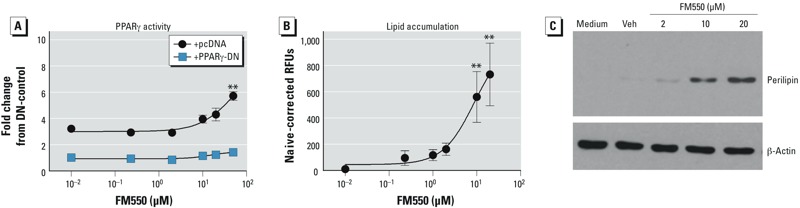
Reporter and *in vitro* differentiation analyses of PPARγ activation by FM550. (*A*) Cos-7 cells were transiently transfected with human *PPARG1* and PPRE x3-TK-luc, with either pcDNA3 or PPARγ-DN vectors. Transfected cultures were treated with vehicle (Veh; DMSO, reported as 10^–2^ μM) or FM550 (0.1–20 μg/mL; 0.2–50 μM) and incubated for 24 hr; reporter activation was assessed by luciferase expression and normalized by eGFP fluorescence. (*B*–*C*) Confluent BMS2 cultures were treated with Veh (DMSO, reported as 10^–2^ μM) or FM550 (0.1–10 μg/mL; 0.2–20 μM), and lipid accumulation (*B*) and perilipin expression (*C*) were quantified after 7 days. (*A,B*) Data are presented as mean ± SE of 3–7 biological replicates. (*C*) Data are representative of 3–7 biological replicates. *p < 0.05, and **p < 0.01, by ANOVA and Dunnett’s multiple comparisons test, compared with Veh treatment.

*Assessment of PPAR*γ *activation by FM550*. To test the hypothesis that FM550 can activate PPARγ transcriptional activity and directly induce adipogenesis, we began by investigating the ability of FM550 to activate PPARγ-driven reporter activity. Cos7 cells were transfected with human *PPARG1* and *RXRA* expression vectors and a PPRE-driven reporter construct and treated with vehicle or FM550. FM550 significantly induced PPARγ-driven reporter activity at concentrations ≥ 10 μg/mL (20 μM), with an EC_50_ (concentration required to produce 50% of maximal effect) of 47 μM (Figure 1A). The maximal FM550-induced activity of 5.7 ± 0.3-fold was less than the activity induced by a maximally efficacious concentration of rosiglitazone (1 μM; 11.3 ± 0.9-fold; EC_50_ of 0.02 μM; see Supplemental Material, Figure S2A). The specificity of reporter activity was determined by co-transfecting Cos7 cells with a DN-PPARγ expression vector. The presence of DN-PPARγ significantly reduced FM550-induced reporter expression (Figure 1A).

To examine the ability of FM550 to induce adipocyte differentiation, BMS2 cells were grown to confluence and then treated with vehicle or FM550. FM550 significantly induced lipid accumulation at concentrations ≥ 5 μg/mL (10 μM) (Figure 1B). The maximal FM550-induced lipid accumulation of 732 ± 138 RFUs was less than the lipid accumulation induced by a maximally efficacious concentration of rosiglitazone (1 μM; 1,043 ± 45 RFUs; see Supplemental Material, Figure S2B). To confirm that FM550 stimulated terminal adipocyte differentiation, BMS2 cells were assessed for expression of the adipocyte-specific protein perilipin (Greenberg et al. 1991). Treatment with FM550 resulted in increased expression of perilipin (Figure 1C). The results indicate that FM550 contains a component or components capable of activating PPARγ and stimulating adipocyte differentiation.

**Figure 2:**
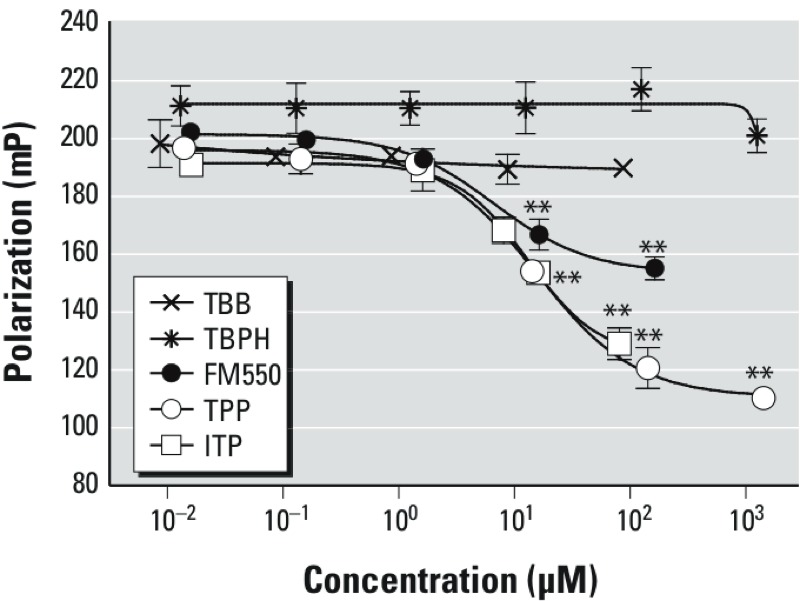
Determination of PPARγ ligand binding affinities of FM550 and its components. FM550 (0.01–70 μg/mL; 0.02–160 μM), TPP (0.01–1,400 μM), TBB (0.009–90 μM), TBPH (0.12–1,200 μM), and ITP (0.01–28 μg/mL; 0.02–80 μM) were tested in the PolarScreen™ PPARγ-competitor assay. IC_50_ values and dissociation constants were calculated as described in “Materials and Methods.” Data are presented as mean ± SE of three technical replicates and are representative of two independent experiments. **p < 0.01, by ANOVA and Dunnett’s multiple comparisons test, compared with the lowest concentration.

*Computational and* in vitro *analyses of PPAR*γ *binding by components of FM550*. To test the hypothesis that components of FM550 are PPARγ ligands, we assessed the ability of FM550 and its components to bind with the PPARγ LBD (Figure 2). We found that FM550 could competitively bind with the PPARγ LBD in a dose-dependent manner (IC_50_ = 400 μM; *K*_d_ = 210 μM). The brominated components of FM550, TBB, and TBPH did not demonstrate any binding over the concentration range tested. In contrast, TPP was found to be a ligand of PPARγ (IC_50_ = 38 μM; *K*_d_ = 20 μM). ITP showed an ability similar to that of TPP to compete for PPARγ binding (IC_50_ = 60 μM; *K*_d_ = 32 μM). In comparison, rosiglitazone competed for PPARγ binding with an IC_50_ of 0.23 μM and a *K*_d_ of 0.12 μM (see Supplemental Material, Figure S2C). Because ITP contains approximately 40% TPP (measured in the laboratory of H.M.S.), TPP was likely a significant contributor to PPARγ binding by the ITP mixture; however, the other isopropylated phosphate isomers in this mixture may also effectively bind to PPARγ-LBD.

**Figure 3:**
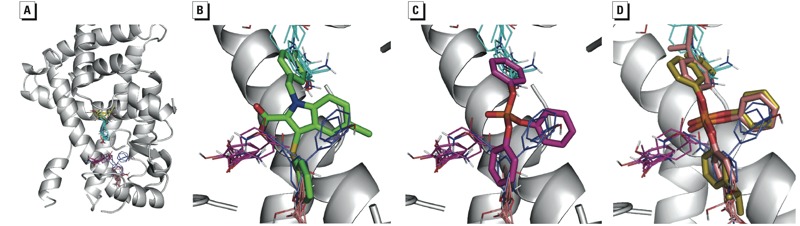
Computational analysis of TPP and ITP interactions with the PPARγ LBD. (*A*) Mapping of the PPARγ LBD structure (PDB ID 2q5s). The protein is shown as a gray cartoon, and representatives of probe clusters within the various consensus clusters are shown as lines. The color code is as follows: CC1 (27 probe clusters), cyan; CC2 (17 probe clusters), magenta; CC3 (16 probe clusters), yellow; CC4 (13 probe clusters), salmon; and CC5 (9 probe clusters), blue. To allow the probe clusters to be seen, some parts of the PPARγ were removed. (*B*) Close-up of the mapping results. The bound pose of the partial agonist nTZDpa (shown as green sticks) is superimposed for reference; the three rings of nTZDpa are bound at the hot spots, defined by the consensus clusters CC1, CC4, and CC5, respectively, whereas the carboxylic acid of nTZDpa orients toward CC2. (*C*) Best docked pose of TPP (shown as magenta sticks); the rings in TPP interact with the same three hot spots at CC1, CC4, and CC5. (*D*) Best docked poses for two isopropylated derivatives of TPP.

The interaction of TPP and ITP with the PPARγ LBD was assessed computationally. In a previous study, the FTMap solvent mapping program (Brenke et al. 2009) was used to identify two main ligand binding regions within PPARγ’s large binding site (Sheu et al. 2005). The first of these binding regions is located at the polar headgroup of thiazolidinediones (TZDs), interacting with the H12 helix of the LBD, and the second is between the distal end of the TZDs and the entrance of the ligand binding site. Because the first region is too narrow for the binding of TPP, we focused on the second site, which is known to bind selective partial agonists (Bruning et al. 2007). Mapping of the latter region shows four binding hot spots (Figure 3A). As shown in Figure 3B, the locations of these hot spots are in good agreement with the positions of the three rings and the carboxylic acid group in several selective partial agonists, such as 5-chloro-1-(4-chlorobenzyl)-3-(phenylthio)-1h-indole-2-carboxylic acid (also called nTZDpa), that bind at this site and activate PPARγ using an H12-independent mechanism (Bruning et al. 2007). In the best docked mode of TPP, the three rings overlap with the three hot spots, which also interact with the rings of nTZDpa (Figure 3C). Ligands that bind to a site generally overlap well with binding hot spots (Hall et al. 2012); therefore, on the basis of our results, we are convinced that TPP binds to PPARγ in a manner very similar to the binding of selective partial agonists. The hot spots extend beyond the rings, and thus PPARγ very likely also binds the various isopropylated derivatives of TPP (Figure 3D).

**Figure 4:**
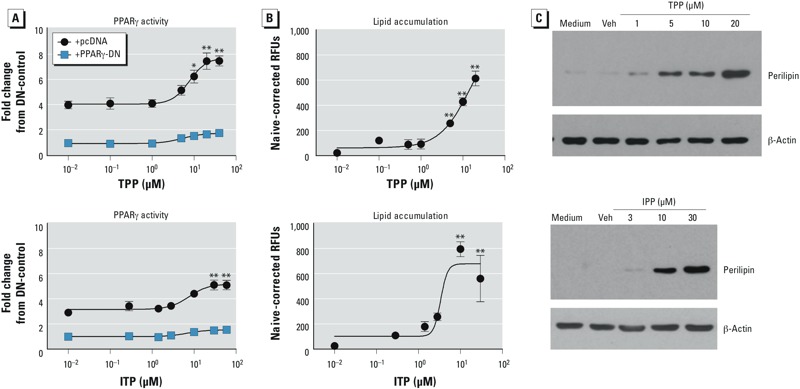
Reporter and *in vitro* differentiation analyses of PPARγ activation by TPP (top) and ITP (bottom). (*A*) Cos-7 cells were transiently transfected with human *PPARG1* and PPRE x3-TK-luc, with either pcDNA3 or PPARγ-DN vectors. Transfected cultures were treated with vehicle (Veh; DMSO, reported as 10^–2^ μM), TPP (0.1–40 μM), or ITP (0.1–10 μg/mL; 0.3–60 μM) and incubated for 24 hr; reporter activation was assessed by luciferase expression and normalized by eGFP fluorescence. (*B*–*C*) Confluent BMS2 cultures were treated with Veh (DMSO, reported as 10^–2^ μM), TPP (0.1–20 μM), or ITP (0.1–10 μg/mL; 0.3–30 μM), and lipid accumulation (*B*) and perilipin expression (*C*) were quantified after 7 days. (*A,B*) Data are presented as mean ± SE of 3–7 biological replicates. (*C*) Data are representative of 3–7 biological replicates. *p < 0.05, and **p < 0.01, by ANOVA and Dunnett’s multiple comparisons test, compared with Veh treatment.

*Assessment of PPAR*γ *activation by the organophosphate components of FM550*. As with FM550, we examined the ability of TPP and ITP to activate PPARγ-driven reporter expression and induce adipogenesis. In the PPARγ/RXRα Cos7 cell reporter assay, TPP significantly induced PPARγ-driven reporter activity at concentrations ≥ 10 μM, with an EC_50_ of 8 μM and maximal activity of 7.5 ± 0.7-fold (Figure 4A), and ITP significantly induced PPARγ-driven reporter activity at concentrations ≥ 10 μg/mL (30 μM), with an EC_50_ of 8 μM and maximal activity of 5.1 ± 0.6-fold (Figure 4A). TPP and ITP are less potent and efficacious than rosiglitazone (1 μM; 11.3 ± 0.6-fold; EC_50_ of 0.02 μM; see Supplemental Material, Figure S2A). In the BMS2 adipogenesis assay, TPP significantly induced lipid accumulation at concentrations ≥ 5 μM with a maximal lipid accumulation of 614 ± 60 RFUs (Figure 4B), and ITP significantly induced lipid accumulation at concentrations ≥ 1 μg/mL (3 μM), with a maximal lipid accumulation of 796 ± 60 RFUs (Figure 4B); these lipid accumulations were lower than the lipid accumulation induced by a maximally efficacious concentration of rosiglitazone (1 μM; 1043 ± 45 RFUs; see Supplemental Material, Figure S2B). That TPP and ITP induced terminal adipocyte differentiation was confirmed by the observation that both compounds induced the expression of perilipin (Figure 4C). The results indicate that TPP and ITP are PPARγ ligands that can induce adipocyte differentiation.

**Figure 5:**
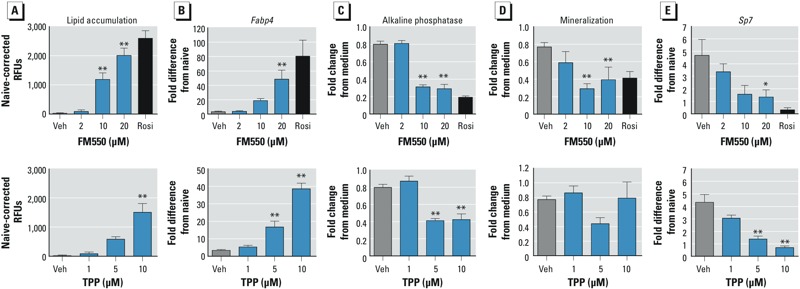
Assessment of effects of FM550 (top) and TPP (bottom) on osteogenesis *in vitro*. Primary bone marrow cultures were established, and osteogenesis was initiated with the addition of ascorbate, β-glycerol phosphate, insulin, and dexamethasone, except for naive wells. Cells were treated with vehicle (Veh; DMSO), FM550 (0.1–10 μg/mL; 0.2–20 μM), TPP (0.1–10 μM), or rosiglitazone (Rosi; 0.1 μM) and cultured for 7 days (gene expression) or 12 days (phenotype). (A) Lipid accumulation. (B) *Fabp4* mRNA expression. (C) Alkaline phosphatase activity. (D) Mineralization. (E) *Sp7* mRNA expression. Data are presented as mean ± SE of 4–6 independent bone marrow preparations. *p < 0.05, and **p < 0.01, by ANOVA and Dunnett’s multiple comparisons test, compared with Veh treatment.

*Analysis of effects of FM550 and TPP on bone differentiation* in vitro. To test the hypothesis that FM550 and TPP are negative regulators of bone formation, we examined the effect of FM550 and TPP on adipogenic and osteogenic differentiation in primary bone marrow cultures prepared from female C57BL/6J mice. Established bone marrow cultures were induced to undergo osteogenesis and treated with vehicle, FM550, TPP, or rosiglitazone. FM550 induced significant lipid accumulation at a concentration of 5 μg/mL (10 μM), and TPP induced lipid accumulation at a concentration of 10 μM (Figure 5A). Activation of PPARγ by FM550 and TPP was reflected in the significantly increased mRNA expression of *Fabp4*, a PPARγ-target gene (Tontonoz et al. 1994) (Figure 5B). Although FM550 significantly suppressed both alkaline phosphatase activity and mineralization at 5 μg/mL (10 μM), TPP significantly suppressed only alkaline phosphatase activity (Figure 5C,D). Suppression of the transcriptional program of Runx2, the master regulator of osteogenesis, by FM550 and TPP was indicated by the significant decrease in mRNA expression of *Sp7*, a Runx2-target gene (Bonewald 2011) (Figure 5E). Taken together, these results suggest that FM550 and TPP can divert MSC differentiation away from osteogenesis and toward adipogenesis and that the FM550 mixture, as a whole, may have a greater effect than TPP.

*Estimated indoor exposure of children to TPP*. We used a recently published screening-level exposure model for SVOCs (Little et al. 2012) to estimate the contribution of dust to TPP exposure. The geometric mean concentration of the log-normally distributed TPP in 50 Boston house dust samples was 7.36 μg/g (range, < 0.15 to 1,800 μg/g) (Stapleton et al. 2009). Applying Equations 2 and 3 to the geometric mean dust concentration estimated an indoor vapor concentration of 3.4 ng/m^3^ (85% of total air concentration) and a *y*_0_ of 7.2 ng/m^3^. Table 1 shows the estimated exposure to TPP by route for 3-year-old children based on these indoor concentrations. The overall estimated exposure was 0.5 μg/day, with 87% due to dust ingestion. Use of the maximum TPP dust concentration of 1,800 μg/g from Stapleton et al. (2009) yields an estimated total indoor exposure for children of 120 μg/day with the same breakdown by exposure pathway.

**Table 1:** Estimated exposure of 3-year-old children to TPP (based on geometric mean dust concentrations from 50 Boston homes).

Exposure pathway	Exposure (μg/day)	Percent of total
Inhalation (vapor)	0.028	5.5
Inhalation (particles)	0.005	0.9
Ingestion (dust)	0.44	87.0
Dermal sorption (from vapor)	0.031	6.1
Total	0.50	100.0
Assuming an inhalation rate of 8.9 m^3^/day, a dust ingestion rate of 60 mg/day, exposed skin surface area of 0.61 m^2^, an exposure duration of 21.9 hr/day (Little et al. 2012).		

## Discussion

Obesity and osteoporosis are two of the most pervasive chronic health-care problems in the industrialized world. Grun et al. (2006) suggested that exposure to environmental obesogens may play a role in the obesity epidemic. That a growing number of environmental PPARγ agonists are being shown to not only enhance adipocyte differentiation but also suppress osteogenesis suggests that environmental toxicants may contribute to loss of bone health. Patisaul et al. (2013) reported that prenatal and postnatal exposure to FM550 resulted in obesity and increased anxiety and early puberty in rats. In the present study, we observed that the flame retardant mixture FM550 contains PPARγ ligands and stimulates adipogenesis, and we identified a novel PPARγ ligand, TPP, that modified MSC differentiation.

FM550 binds to the human PPARγ LBD, activates human PPARγ1 transcriptional activity, and stimulates adipogenesis. PPARγ is the master regulator of adipocyte differentiation (Tontonoz and Spiegelman 2008), and its activation by thiazolidinedione drugs increases fat mass and weight gain (Carmona et al. 2005). Taken together, these results suggest that the increased fat mass observed in rats treated perinatally with FM550 (Patisaul et al. 2013) was a result of PPARγ activation.

FM550 is a mixture of brominated components (TBB and TBPH) and triaryl phosphates (TPP and ITP). Previous analyses of the brominated components of FM550 showed that a brominated metabolite of TBPH—mono-(2-ethylhexyl) tetrabromophthalate—induced adipocyte differentiation in NIH 3T3L1 cells and activated PPARα- and PPARγ-mediated gene transcription *in vitro* (Springer et al. 2012). However, TBPH is minimally metabolized into the monoester *in vivo* and *in vitro* (Patisaul et al. 2013; Roberts et al. 2012). In the present study, we observed that the brominated components of FM550 did not bind PPARγ. However, both TPP and ITP (which contains 40% TPP) bound to the human PPARγ LBD, interacted with binding hot spots within the LBD, activated PPARγ1 transcriptional activity, and stimulated adipogenesis. Interestingly, the tri-substituted OPFRs have a structural similarity to organotins, a class of compounds for which the tri-substitution is known to be important for obesogenic activity (Grun et al. 2006). Thus, TPP is likely a significant contributor to the obesogenic activity of FM550.

Osteoporosis has been referred to as “obesity of the bone” (Rosen and Bouxsein 2006). PPARγ plays a crucial role in MSC differentiation, both by activating adipogenic differentiation and by suppressing osteoblast differentiation (Akune et al. 2004). Accordingly, treatment with the therapeutic PPARγ ligand rosiglitazone, either *in vivo* or in *in vitro* bone marrow MSC models, results in increased expression and activity of PPARγ and a concomitant decrease in Runx2 expression and activity (Ali et al. 2005; Rzonca et al. 2004). In the present study we observed that FM550 and TPP could divert bone marrow MSC differentiation away from bone formation and toward adipocyte differentiation. These findings point to the need for serious consideration of whether environmental PPARγ ligands (e.g., TPP, phthalates, organotins) also have detrimental effects on bone health by accelerating osteoporosis or enhancing osteoporotic pathology.

The organophosphate components of FM550 had significant biological activity at concentrations of 1–10 μM. The number of assessments of human exposure to OPFRs is growing; however, few studies of human body burden exist. Analyses of OPFRs in human milk have shown that TPP is a common contaminant and that levels of total OPFRs can reach 600 ng/g lipid (Kim et al. 2014). For comparison, a molar concentration of OPFRs of 0.1 μM can be estimated by assuming a concentration of 41 g lipid/L milk (Kent et al. 2006) and an average OPFR molecular weight of 300 g/mol (the molecular weight of TPP is 326 g/mol).

Little information is available on indoor exposure of children to TPP, and the relative importance of dust ingestion for TPP is unknown. Most previous estimates of exposure to flame retardants such as pentaBDE suggest that dust ingestion is much more important to exposure than inhalation, particularly for children. However, the vapor pressure of TPP is one to two orders of magnitude higher than those of BDE-47 and BDE-99, major components of pentaBDE (Weschler and Nazaroff 2008). Furthermore, there have been few estimates of dermal absorption of flame retardants from vapor. Nevertheless, our modeling suggests that dust ingestion accounts for about 87% of exposure in 3-year-old children, with the remainder roughly split between inhalation and dermal absorption of vapor. Meeker et al. (2013) reported no significant association between diphenyl phosphate in urine of adult males and TPP in dust from their homes. The authors listed several potential explanations, including logistics of the sample collections, exposure in other microenvironments, and exposure via inhalation. Our modeling suggests that exposure to house dust is the major route of exposure for young children at home, with inhalation and dermal absorption of TPP playing small but non-negligible roles. These estimates rely on a number of assumptions (e.g., dust ingestion rates are relatively uncertain), and other exposure routes are not included (e.g., dermal absorption following contact with dust, surface films, or personal care products) (Little et al. 2012). Hence, additional research on exposure to TPP is needed. Use of the maximum TPP dust concentration from the 50 Boston homes yielded an exposure estimate for children of 120 μg/day or approximately 9 μg/kg BW/day of TPP. For comparison, Patisaul et al. (2013) exposed dams to 0.1 and 1 mg/day FM550, or approximately 0.25–2.5 mg/kg BW/day, of which TPP represented approximately 10–20%. Although there are several difficulties in making these comparisons, our results suggest that additional lower-dose toxicological studies are needed to determine whether current exposure to TPP and other PPARγ ligands pose health risks.

## Conclusions

Results from this study support the conclusion that the alternative flame retardant mixture FM550 may be obesogenic because it contains a PPARγ ligand(s). The likely mediator of the adipogenic effect of FM550 is TPP, as TPP binds PPARγ, activates PPARγ-mediated transcription and induced adipogenesis. That FM550 and TPP are adipogenic has implications for both development of obesity and loss of bone health in humans.

## Supplemental Material

(2 MB) PDF
