# Supplementary material for: Ligand Binding and Activation of PPARγ by Firemaster® 550: Effects on Adipogenesis and Osteogenesis in Vitro
Source: Environ Health Perspect. 2014 Jul 25;122(11):1225–32. doi: 10.1289/ehp.1408111 (PMC4216168; doi:10.1289/ehp.1408111)
Supplement: (2 MB) PDF [file ehp.1408111.s001.508.pdf]

Environmental Health Perspectives doi:10.1289/ehp.1408111

Supplemental Material

Ligand Binding and Activation of PPARγ by Firemaster® 550: Effects on Adipogenesis and Osteogenesis *in Vitro*

Hari K. Pillai, Mingliang Fang, Dmitri Beglov, Dima Kozakov, Sandor Vajda, Heather M. Stapleton, Thomas F. Webster, and Jennifer J. Schlezinger

Table of Contents **Page**

Supplemental methods 2

Computational analysis of ligand binding to PPARγ 2

Table S1. Estimation of FM550 and ITP molecular weights 4

Table S2. Estimation of FM550 and ITP molar concentrations 5

Figure S1. Assessment of toxicity of FM550, ITP and TPP following short, medium and long term exposures 7

Figure S2. PPARγ-mediated and adipogenic responses to rosiglitazone. 8

References 9

Supplemental methods

Computational analysis of ligand binding to PPARγ

The computational analysis of TPP and ITP binding was enabled by the availability of many X-ray crystal structures of the PPARγ LBD in the Protein Data Bank (Berman et al. 2000), some of them solved in complex with molecules that are fairly similar to compounds with flame retardant properties. The first step of the analysis was determining the binding hot spots of the PPARγ LBD using the computational solvent mapping algorithm FTMap (Brenke et al. 2009). The method places small molecular probes of various sizes and shapes on a dense grid around the protein, finds favorable positions using empirical energy functions, clusters the conformations, and ranks the clusters on the basis of the average energy. All ligands and crystallographic water molecules are removed prior to mapping. The regions that bind multiple low energy probe clusters, called consensus cluster (CC) sites, identify the binding hot spots. The hot spots are ranked in terms of the number of overlapping probe clusters contained. The consensus cluster with the highest number of probe clusters is ranked first as CC1; nearby consensus clusters within 7 Å center-to-center distance are also joined with CC1 to form the predicted ligand binding site (Ngan et al. 2012).

The mapping results were used in two different ways for facilitating the docking of ligands to PPARγ LBD. First, a box with 4 Å padding was created around the predicted binding site. The docking of ligands was carried out using the docking program AutoDock Vina 1.1.0 (Trott and Olson 2010) with standard settings and restricting consideration to the selected box. The 10 lowest energy binding poses were retained for each ligand. Second, the selection of the most likely pose was based on the atom densities calculated from the mapping results (Kozakov et al.

2011). Probe atom density was defined at each point of the binding site as the total number of probe atoms within a 1.25 Å radius (Hall et al. 2010). We considered each retained ligand pose separately and summed the atomic densities for all heavy atoms, resulting in a measure of overlap between the pose and the probe density. The poses were ranked on the basis of this overlap measure, and the pose with the best overlap was selected as the most likely binding mode (Kozakov et al. 2011). The complex was refined by minimizing the CHARMM energy function (Brooks et al. 1983).

Table S1. Estimation of FM550 and ITP molecular weights.

Exposure	% of Mixture	Molecular weight (g/mol)	Contribution to mixture molecular weight
**FM550**[a]			
ITP	45	352.8	158.8
TBB	30	550.9	165.3
TPP	17	326.3	55.5
TBPH	8	706.1	56.5
Estimated molecular weight (g/mol)			436.0
**ITP**[b]			
TPP	40	326.3	130.5
Mono-ITP	42.9	357.3	153.3
Di-ITP	13.4	388.3	52.0
Tri-ITP	3.2	450.3	14.4
Tetra-ITP	0.5	512.3	2.6
Estimated molecular weight (g/mol)			352.8

[a]Mixture fractions based on (McGee et al. 2013). [b]Mixture fractions based on (Klosterhaus et al. 2009).

Table S2. Estimation of FM550 and ITP molar concentrations. Using the molecular weights determined in Table S1, concentrations of FM550 and ITP were estimated. Dose solutions were prepared based on the µg/ml concentration as indicated.

Exposure	µg/ml	µM	M
FM550	0.1	0.2	2E-07
FM550	0.5	1	1E-06
FM550	1	2	2E-06
FM550	5	10	1E-05
FM550	10	20	2E-05
FM550	20	50	5E-05
FM550	40	90	9E-05
ITP	0.1	0.3	3E-07
ITP	0.5	1	1E-06
ITP	1	3	3E-06
ITP	5	10	1E-05
ITP	10	30	3E-05
ITP	20	60	6E-05
ITP	40	100	1E-04
TPP	0.03	0.1	1E-07
TPP	0.3	1	1E-06
TPP	1.6	5	5E-06
TPP	3.3	10	1E-05
TPP	6.5	20	2E-05
TPP	13.0	40	4E-05

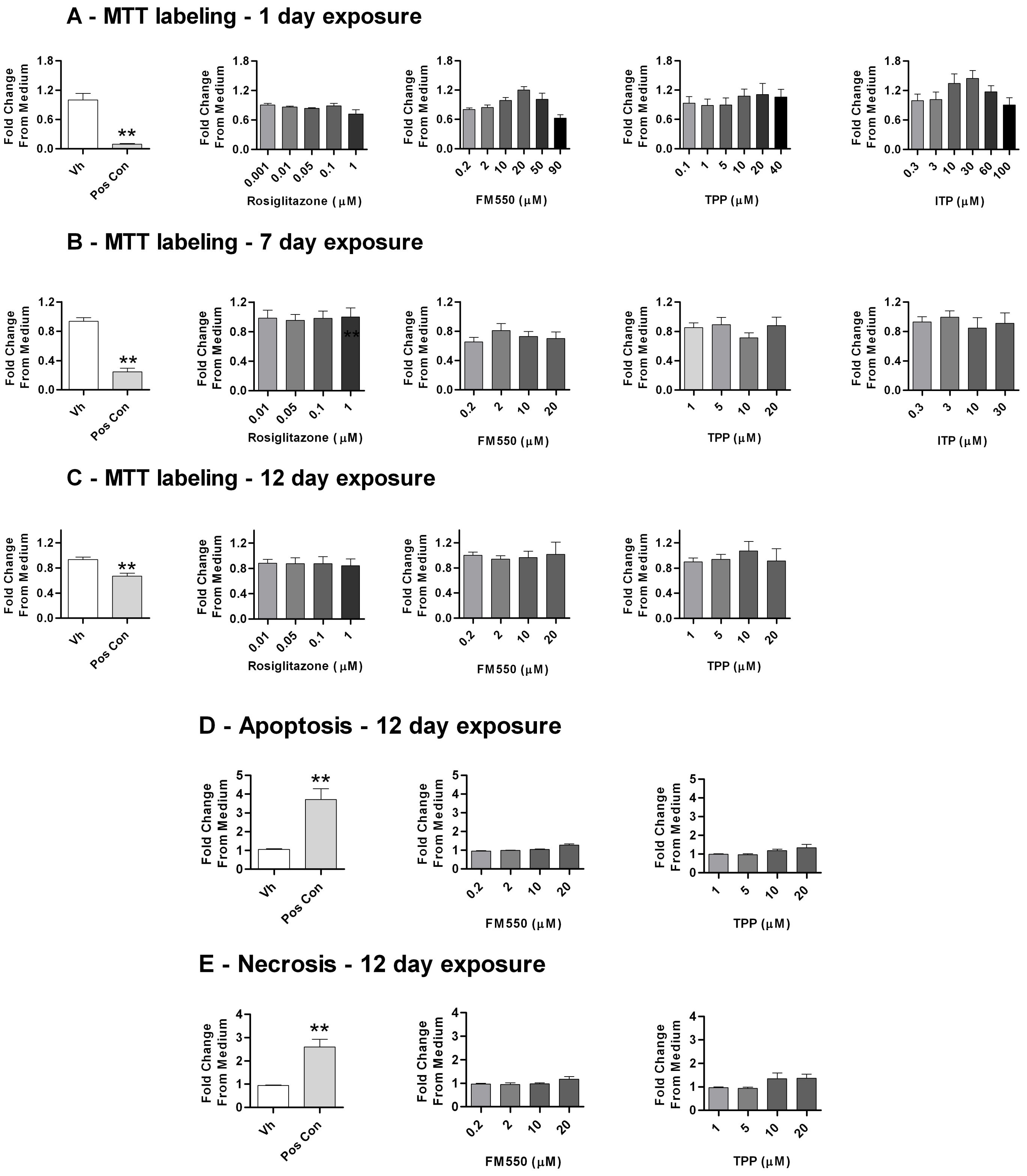

A - MTT labeling - 1 day exposure
Fold Change From Medium
Vh
Pos Con
**
Rosiglitazone (μM)
FM550 (μM)
TPP (μM)
ITP (μM)
B - MTT labeling - 7 day exposure
Fold Change From Medium
Vh
Pos Con
**
Rosiglitazone (μM)
FM550 (μM)
TPP (μM)
ITP (μM)
C - MTT labeling - 12 day exposure
Fold Change From Medium
Vh
Pos Con
**
Rosiglitazone (μM)
FM550 (μM)
TPP (μM)
D - Apoptosis - 12 day exposure
Fold Change From Medium
Vh
Pos Con
**
FM550 (μM)
TPP (μM)
E - Necrosis - 12 day exposure
Fold Change From Medium
Vh
Pos Con
**
FM550 (μM)
TPP (μM)

Figure S1. Assessment of toxicity of FM550, ITP and TPP following short (A), medium (B) and long (C-E) term exposures. Confluent BMS2 cultures were treated with Vh (DMSO), FM550 (0.1-40 μg/ml; 0.2-90 μM), TPP (0.1-40 μM), or ITP (0.1-40 μg/ml; 0.3-100 μM) or rosiglitazone (0.001-1 μM) for 24 hrs (A) and 7 days (B) or with DMSO, rosiglitazone, FM550 or TPP for 12 days (C-E). Medium was changed and the cultures were redosed as described in the Methods. A treatment with 1 (24 hr experiment) or 2-4 μM (7-12 day experiments) tributyltin for 2-3 hrs was used as a positive control. (A-C) Cellularity was assessed by analyzing MTT labeling. (D) Apoptosis was assessed by analyzing caspase-3 activity. (E) Necrosis was assessed by analyzing dead cell protease release. Data are presented as means ± SE (n=4-7). The positive control induced a statistically significant difference in all assays (**$p<0.01$, Student's T Test). No statistically significant differences were observed in experimental samples (ANOVA).

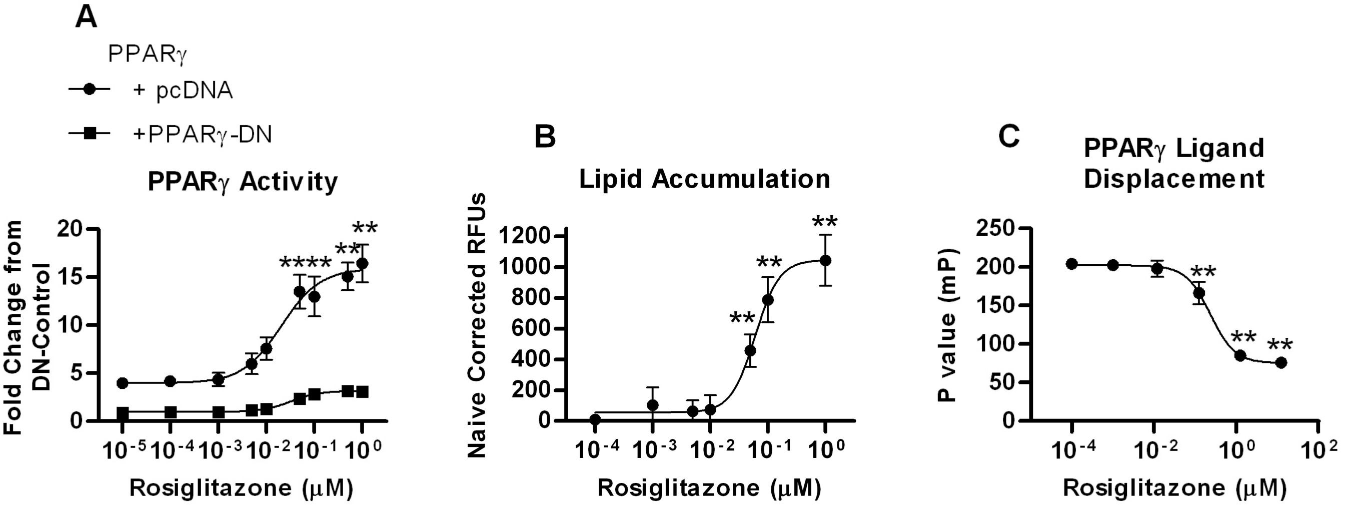

Figure S2. PPARγ-mediated and adipogenic responses to rosiglitazone. (A) Cos-7 cells were transiently transfected with human *PPARG1* and PPRE x3-TK-luc, with either pcDNA3 or PPARγ-DN vectors. Transfected cultures received no treatment (Naïve) or were treated with Vh (DMSO, reported as 10^{-5} μM) or rosiglitazone (0.0001-1 μM) and incubated for 24 hrs. Reporter activation was assessed by luciferase expression and normalized by eGFP fluorescence. Data are presented as means ± SE (n=4). Statistically different from Vh-treated (**p<0.01, ANOVA, Dunnett's). (B) Confluent BMS2 cultures were treated with Vh (DMSO, reported as 10^{-4} μM) or rosiglitazone (0.001-1 μM), and lipid accumulation was quantified after 7 days. Data are presented as means ± SE (n=3). Statistically different from Vh-treated (**p<0.01, ANOVA, Dunnett's). (C) The PolarScreen™ PPARγ-competitor assay was used to determine binding affinities. Rosiglitazone (0.00012-12 μM) was applied to the assay in DMSO. Data are presented as means ± SE (n=3). Statistically different from lowest concentration (**p<0.01, ANOVA, Dunnett's).

References

Berman HM, Westbrook J, Feng Z, Gilliland G, Bhat TN, Weissig H, et al. 2000. The Protein Data Bank. Nucleic Acids Res 28:235−242.

Brenke R, Kozakov D, Chuang GY, Beglov D, Hall D, Landon MR, et al. 2009. Fragment-based identification of druggable 'hot spots' of proteins using Fourier domain correlation techniques. Bioinformatics 25:621−627.

Brooks BR, Bruccoleri RE, Olafson BD, States DJ, Swaminathan S, Karplus M. 1983. CHARMM - a program for macromolecular energy, minimization, and dynamics calculations. J Comp Chem 4:187−217.

Hall DR, Ngan CH, Zerbe BS, Kozakov D, Vajda S. 2010. Hot spot analysis for driving the development of hits into leads in fragment-based drug discovery. J Chem Inf Model 52:199−209.

Klosterhaus S, Konstantinov A, Davis E, Klein J, Stapleton. 2009. Characterization of organophosphorous chemicals in a PentaBDE replacement mixture and their detection in biosolids. In: 11th Annual Workshop on Brominated Flame Retardants. Ottawa, Ontario. CA.

Kozakov D, Hall DR, Chuang GY, Cencic R, Brenke R, Grove LE, et al. 2011. Structural conservation of druggable hot spots in protein-protein interfaces. Proc Natl Acad Sci U S A 108:13528−13533.

McGee SP, Konstantinov A, Stapleton HM, Volz DC. 2013. Aryl phosphate esters within a major PentaBDE replacement product induce cardiotoxicity in developing zebrafish embryos: potential role of the aryl hydrocarbon receptor. Toxicol Sci 133:144−156.

Ngan CH, Hall DR, Zerbe B, Grove LE, Kozakov D, Vajda S. 2012. FTSite: high accuracy detection of ligand binding sites on unbound protein structures. Bioinformatics 28:286−287.

Trott O, Olson AJ. 2010. AutoDock Vina: improving the speed and accuracy of docking with a new scoring function, efficient optimization, and multithreading. J Comp Chem 31(2): 455−461.
